# Effect of P144® (Anti-TGF-β) in an “In Vivo” Human Hypertrophic Scar Model in Nude Mice

**DOI:** 10.1371/journal.pone.0144489

**Published:** 2015-12-31

**Authors:** Shan Shan Qiu, Javier Dotor, Bernardo Hontanilla

**Affiliations:** 1 Department of Plastic and Reconstructive Surgery, Clínica Universidad de Navarra, Pamplona, Spain; 2 Digna Biotech S.L., Pamplona, Spain; Genomics Institute Novartis Research Foundation, UNITED STATES

## Abstract

**Background:**

Hypertrophic scars are one of the most important complications in surgery due to their cosmetic and functional impairments. Previous studies in tissue fibrotic disorders have shown promising results by inhibiting the biological activity effect of Transforming Growth Factor-beta 1 (TGF-β1). The aim of the current study was to determine the clinical effect of the inhibition of TGF-β1 signaling in human hypertrophic scars implanted in nude mice by topical application of an inhibitor of TGF-β1 (P144®).

**Material and Methods:**

A total of 30 human hypertrophic scars were implanted in 60 nude mice. The animals were divided in two groups, group A (placebo) and group B (treatment). Group C (basal) was considered as the preimplanted scar samples and they were not implanted in the nude mice. After the shedding period, topical application of a lipogel containing placebo (group A) or P144 (group B) was daily administered during two weeks. The animals were sacrificed upon completion of the study. Total area, thickness and collagen fibers area were measure and compared across all groups. Immunohistochemistry was also performed in order to quantify collagen type I and type III and elastic fiber expressions present in the dermis.

**Results:**

Successful shedding was achieved in 83,3% of the xenografts. The mean time for shedding was 35±5.4 days. Statistically significant differences were found in the total area, collagen fibers area and thickness between the groups. Increased elastic fibers and decreased collagen I were found in the P144-treated group compared to the basal group.

**Conclusion:**

Topical application of an inhibitor of TGF-β1 may promote scar maturation and clinical improvement of hypertrophic scar morphology features in an “in vivo” model in nude mice after two weeks of treatment.

## Introduction

Abnormal response to injury leading to production of extracellular matrix proteins may lead to the formation of hypertrophic scars and keloids [1]. Wound healing is a complex and multifactorial process, which is carried out through several overlapping dynamic phases and true mechanism is not well established yet [2]. Hypertrophic scars pose a clinically relevant problem as it can be cosmetically disfiguring and functionally debilitating [3,4]. From many studies of burned survivors, hypertrophic scarring seems to be the aspect that affects more deeply their quality of life that in turn can lead to lowered self-esteem and social isolation. The high frequency of hypertrophic scarring found in those patients is not insignificant, ranging from 32 to 72% [5].

A better knowledge of pathogenesis behind abnormal wound healing would lead to better therapeutic strategies [6]. Attempts to develop new treatments should be able to modulate those impaired processes presented in hypertrophic scarring. Many studies have shown that TGF-β plays a critical role in the development of skin fibrotic diseases [7–9]. This molecule is the closet and most representative cytokine that promote fibrosis and scarring in different tissues. It plays a major role in cell differentiation, development and homeostasis [10]. TGF-β1 is secreted by numerous cells, mainly by activated T-cells, macrophages, neutrophils, platelets and precursors in the bone marrow. There are three isoforms with 60–80% homology and they are believed to regulate similar and diverse biological functions: TGF-β1, TGF-β2 and TGF-β3 [2]. TGF-β1 and TGF-β play a fibrogenic role whereas TGF-β3 inhibits this effect [9,11]. TGF-β1 mainly promotes three functions: collagen synthesis and deposition by stimulating fibroblasts, induction of fibroblasts differentiation to myofibroblasts which are provided with alfa- smooth muscle actin (α-SMA) and promotion of extracellular matrix proteins deposition [12]. Its expression is increased during inflammation, cancer or fibrotic induction such as in hypertrophic scars. TGF-β1 signaling pathways function through the TGF-β type I and type II transmembrane serine/threonine protein kinase receptors [2]. Activation of this receptor complex occurs when type II receptor transphosphorylates glycine-serine domain of receptor type I. Then the activated type I kinase is transiently associated with a transmembrane receptor, Smad2/Smad3. When they phosphorylates, the complex binds to Smad4 and then enter the nucleus, where they activate transcription of target genes such as α-SMA and collagen I [13,14]. According to previous studies [7], there is a third receptor, the type III. This is able to activate receptor I and II by phosphorylation whereas its inhibition suppresses the activity of these two receptors. Several studies have recently shown the effect of an inhibitor of TGF-β1 (P144®; Digna Biotech. Spain) in reducing the fibrotic condition of different tissues, such as myocardial [15], liver [16], scleroderma [17] and nerve regeneration [18]. This protein prevents the union of TGF-β1 with its receptors. On the basis of promising results obtained in these studies, we hypothesized that P144 could also have effect on reducing the fibrosis present in hypertrophic scars.

The aim of the present study is to evaluate the clinical effect of topical application of the inhibitor of TGF-β1 (P144®) in an “in vivo” human hypertrophic scars model implanted in nude mice.

## Materials and Methods

The current animal study was conducted in accordance to protocols approved by the Institutional Animal Care and Use Committee and according to the European Communities Council Directive (2010/63UE). Protocols to obtain human tissue samples and participant consents have been approved by the University of Navarra Health Research Ethics Board (CEEA/059-08). All patients gave verbal informed consent to be enrolled in the current study and this information was written in the medical record of each patient. Correction of the hypertrophic scars in these patients was undergone to improve the mobility of the affected area, in cases of a second surgery in the same area using the same scar to approach or as debulking procedure with the attempt to improve the scars cosmesis. Collecting the resected scars for experimental purpose was secondary to the surgery and none of the enrolled patients were asked to undergo the surgery only for research purpose.

The hypertrophic scars were obtained from consecutive female Caucasian patients who underwent elective surgery for scar resection located in the thorax, back or shoulders (n = 30). All patients gave verbal informed consent to be enrolled in the current study and this information was written in the medical record of each patient. Correction of the hypertrophic scars in these patients was undergone to improve the mobility of the affected area, in cases of a second surgery in the same area using the same scar to approach or as debulking procedure with the attempt to improve the scars cosmesis. Collecting the resected scars for experimental purpose was secondary to the surgery and none of the enrolled patients were asked to undergo the surgery only for research purpose. The hypertrophic scars were clinically evidenced from at least 6 months since injury. A total of 60 BALB/c-nu/nu T-deficient nude mice (Harlan Laboratories). The tissue samples were implanted in the interscapular region of nude mice, assigned in two groups. The range of the body weight was 20–25 g and the age was of 4–6 weeks. Surgical procedures in mice were performed under inhaled anesthesia with 2–3% Isoflurane (Abbott S. A. Madrid, Spain) in 100% oxygen. Under sterile conditions and in a laminar airflow cabinet, each tissue sample was split into three pieces of 1x1 cm in size after removing the excessive of subcutaneous fat. Two samples from each scar were implanted onto the back of two different mice (one each from group A and group B) according to the method described by Shetlar [11] and a third piece was used as basal group which was directly fixed in formalin for histological studies (group C). The animals were placed in individual cages provided with positive pressure to prevent contamination. The animals were daily checked by the main researcher and personnel from the animal house. For the first 3 days injection of subcutaneous ketoprofeno 5mg/kg was administered each 12 hours. Enrofloxacina 25kg/kg was administered to prevent postoperative infection during the first 7 days. Group A was treated with placebo and group B was treated with P144®. Peptide P144 (amino acid sequence: TSLDASIIWAMMQNA, MW: 1580.8 Da, encompassing human TGFR-III 730–743) was previously developed by *in silico* predictions software designed by the Department of Internal Medicine from the University of Navarra [16]. In the present work, P144 peptide was manufactured by PolyPeptide Group (Strasbourg, France) as a lyophilized acetate salt compound with purity above 95% (determined by high-performance liquid chromatography and mass spectrometry) and kindly provided by Digna Biotech Ltd. (Madrid, Spain). After the shedding period, all transplanted scars received daily topical application of a lipogel containing either placebo (group A) or P144® (group B) for 2 weeks. The application covered the whole extension of the implanted scar. The composition of the lipogel was: Dimeticone 350 10%, liquid paraffin 40%, clorocresol 0.1%, cetrimide 0.5%, cetoestearilic alcohol 5%, DMSO 0.2% in distilled water until 100%. For group B, P144® at 300μg/ml was added the lipogel.

### Histological analysis

After 44.6–55.4 days (mean time of 47.8 days) mice were sacrificed by cervical dislocation according to the established protocol and whole scars were excised from their back. Biopsy specimens were fixed in 10% formalin for 24 hours and then embedded in paraffin. Paraffin sections were cut into 3μm thick slides and subjected to Hematoxylin & Eosin (H&E) and Masson´s trichrome staining to study the dermal collagen fibers. Total area, collagen fibers area and thickness of the scars were measured using Masson´s trichrome. The morphology of epidermis, presence of skin appendages and presence of collagen fibers bundles presented in superficial and deep dermis were assessed in the histological preparations under microscopic examination (20x magnification).

### Immunocytochemistry for elastic fibers and collagen type I and type III

Antigen retrieval (AR) was performed for immunohistochemical analysis: heat induced AR (anti-fibrillin 1) was applied for 30 min at 95°C in 0,01 M citric acid (pH = 6) in a Pascal pressure chamber (S2800, Dako, Glostrup, Denmark). Proteolytic induced AR was performed using a solution of 20μg/ml proteinase K for 30 min at 37°C (for anti-collagen I) or 4mg/ml pepsin for 1 hour at 37°C (for anti-collagen III). Endogenous peroxidase was blocked with 3% H_2_O_2_ in deionized water for 12 min. and sections were washed in TBS-0.05% Tween 20 (TBS-T). Primary antibodies and their optimal dilutions were: rabbit anti-collagen I (polyclonal, 1:200; 2150–0020, AbD Serotec, Raleigh, NC), mouse anti-collagen III (clone MWD1.1; 1:2000; AM167-5M, BioGenex, Fremont, CA) and mouse anti-fibrillin 1 (clone 11C1.3; 1:100; ab3090, Abcam, Cambridge, UK). Incubations with primary antibodies were performed overnight at 4°C. After rinsing in TBS-T, secondary EnVision peroxidase-labelled goat anti-rabbit (K4011, Dako) or goat anti-mouse (K4007, Dako) was incubated for 30 min at room temperature. Peroxidase activity was revealed using DAB+ substrate chromogen (Dako) as recommended by the manufacturer. Sections were lightly counterstained with Harris hematoxylin. Finally, slides were dehydrated in graded series of ethanol, cleared in xylene and mounted in Cytoseal XYL (8312–4, ThermoFisher Scientific; Walthman, MA).

### Morphometric analysis

Semi-quantitative measurement of the total area, collagen fibers area and the thickness of the scars were performed using digital images acquired with a Zeiss AxioCamICc3 camera (Plan-Neofluar objective with 0.50 NA) at 20x magnification with an AxioImager.M1 microscope (Zeiss, Germany). The quantification was based on collagen fibers stained green with Masson’s Trichrome. Then, AxioVision software was used to form a mosaic of tissue including each picture. Mosaic images were analyzed using an in-house developed plug-in for Fiji (a distribution of Image J) V1.46b. To quantify the scar thickness, an elliptical figure was drawn over the scar image and the shorter axis was taken as its value.

To quantitatively assess the expression level of Collagen I, Collagen III and Fibrillin-1, digital images were acquired using an Aperio Scanscope CS2 (Leica Biosystems) at 20x magnification. Images were analyzed using an in-house developed plug-in for Fiji (a distribution of Image J) V1.48v. Total area of the scars was segmented automatically to obtain total area value measured in pixels^2^. Then images were threshold in order to measure the positive staining area of each marker. The expression level was presented as the positive area ratio. Mean intensity of staining value was also measured for all threshold areas.

### Statistical analysis

Obtained data were compared using the Wilcoxon rank sum test or Kruskal-Wallis test, as appropriate. For the subgroups analyses, T-student was applied. All tests were two-tailed. Values of p< 0.05 were considered significant. SPSS for Windows was the chosen software for performing the analysis (v.15.0; SPSS Inc., Chicago, IL, USA).

## Results

**Fig 1:**
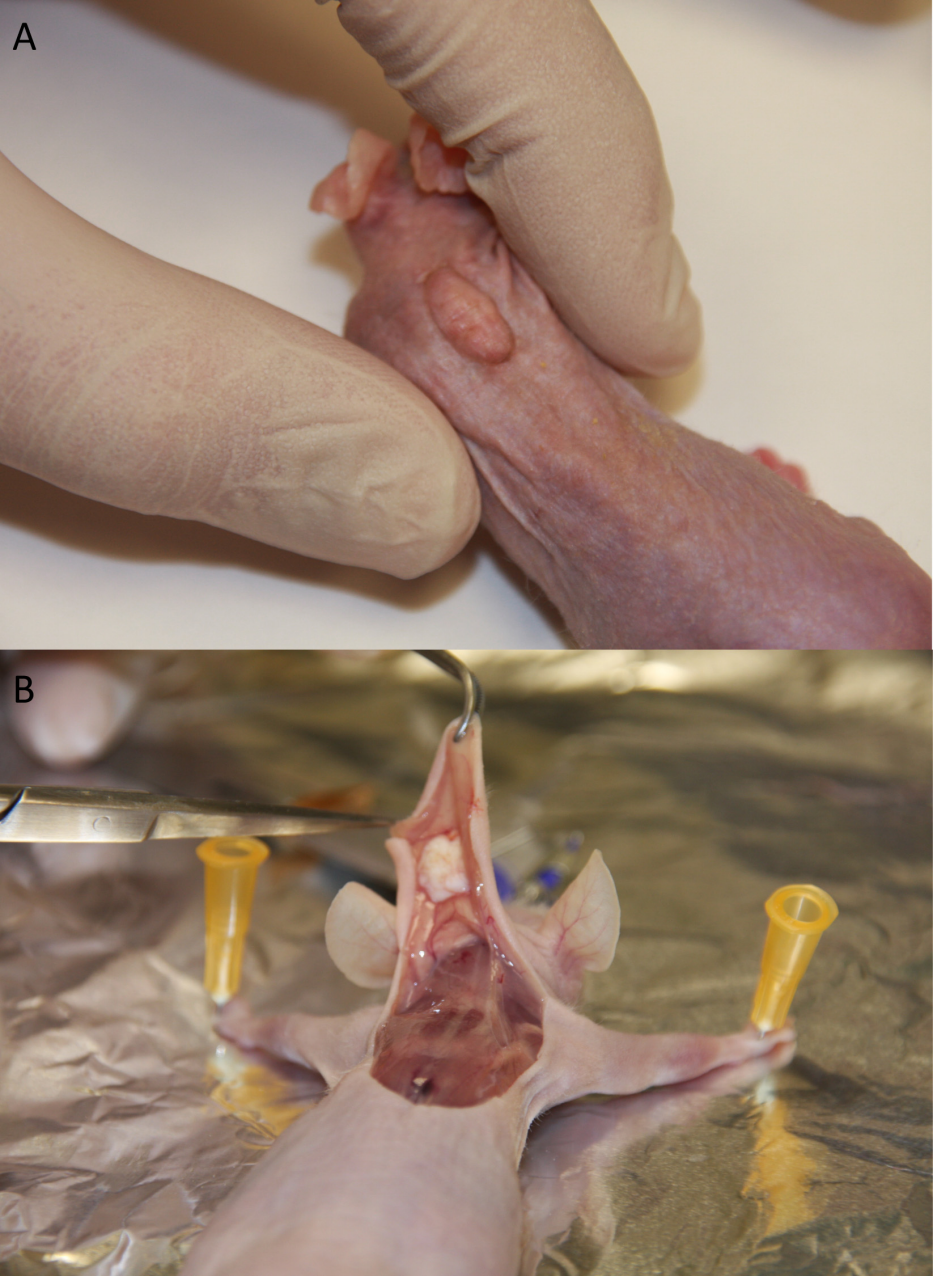
Macroscopic aspect of a hypertrophic scar in a nude mouse. A. Characteristic stiffness of a human hypertrophic scars after 35 days. B. After sacrificing the animal, vessels can be seen coming from the surrounding tissue arrived to nourish the hypertrophic scar.

After completion of grafts healing period, 50 out of 60 grafts were successfully shed (83.33%). Seven mice died likely due to an infection during the post-grafting period. The rest of the animals were sacrificed using cervical dislocation. Five placebo cases developed host vs. graft reaction secondary to surgical sutures. A total of 36 mice (18 pairs) were suitable for testing of P144® effect compared with placebo. The mean time of shedding was 35±5.4 days. Xenografts were shed with the characteristic stiffness of a human hypertrophic scar, elevated, thickened and confined to the site of implantation. Dense collagen bundles confined in the reticular dermis, with absent of human skin appendages confirmed the presence of hypertrophic scarring. Neovascularization could be seen macroscopically as small vessels reaching the grafts from the surrounding tissue of the host. (Fig 1)

Human epidermis and dermis were preserved in all samples and a clear demarcation between human and mouse skin could be seen in most of the specimens. Absent of human skin appendages were seen in any case. In some cases, boundaries of hypertrophic scars were partially embedded under the mouse skin resulting in an image of mouse skin beneath hypertrophic scar. Presence of these images depended on where the paraffin section was placed. This was a phenomenon that occurred at the boundaries of these two tissues but not in the central part. Full thickness scar with intact human epidermis was presented in all samples allowing topical application of P144 in all shed specimens. Changes in the hypertrophic scars histological features suck as total area, collagen fibers area and thickness of the scar parameters were measured in all three experimental groups, and compared using Kruskal-Wallis test. Statistical significance was found for all three parameters when compared to the basal group (Table 1, Table 2 and S1 Table).

**Table 1:** Morphological variables of the hypertrophic scars. Comparison between the groups using Kruskall-Wallis test.

Variable	Group	N	Mean	SD^1^
Total area	Basal	18	3.6	1.97
(cm^2^)	Placebo	18	2.02	1.10
	P144	18	1.68	1.01
Collagen area	Basal	18	2.65	1.01
(cm^2^)	Placebo	18	1.74	1.10
	P144	18	1.49	1.01
Thickness	Basal	18	1.48	0.44
(cm)	Placebo	18	0.99	0.35
	P144	18	0.88	0.36

^1^Standard deviation

**Table 2:** Statistical results in the comparison between subgroups.

Variables	Placebo Vs. P144	Basal Vs. P144	Basal Vs. Placebo
Total area	p = 0.231	p = 0.005	p = 0.006
Collagen area	P = 0.306	P = 0.028	P = 0.022
Thickness	P = 0.170	P = 0.004	P = 0.001

**Fig 2:**
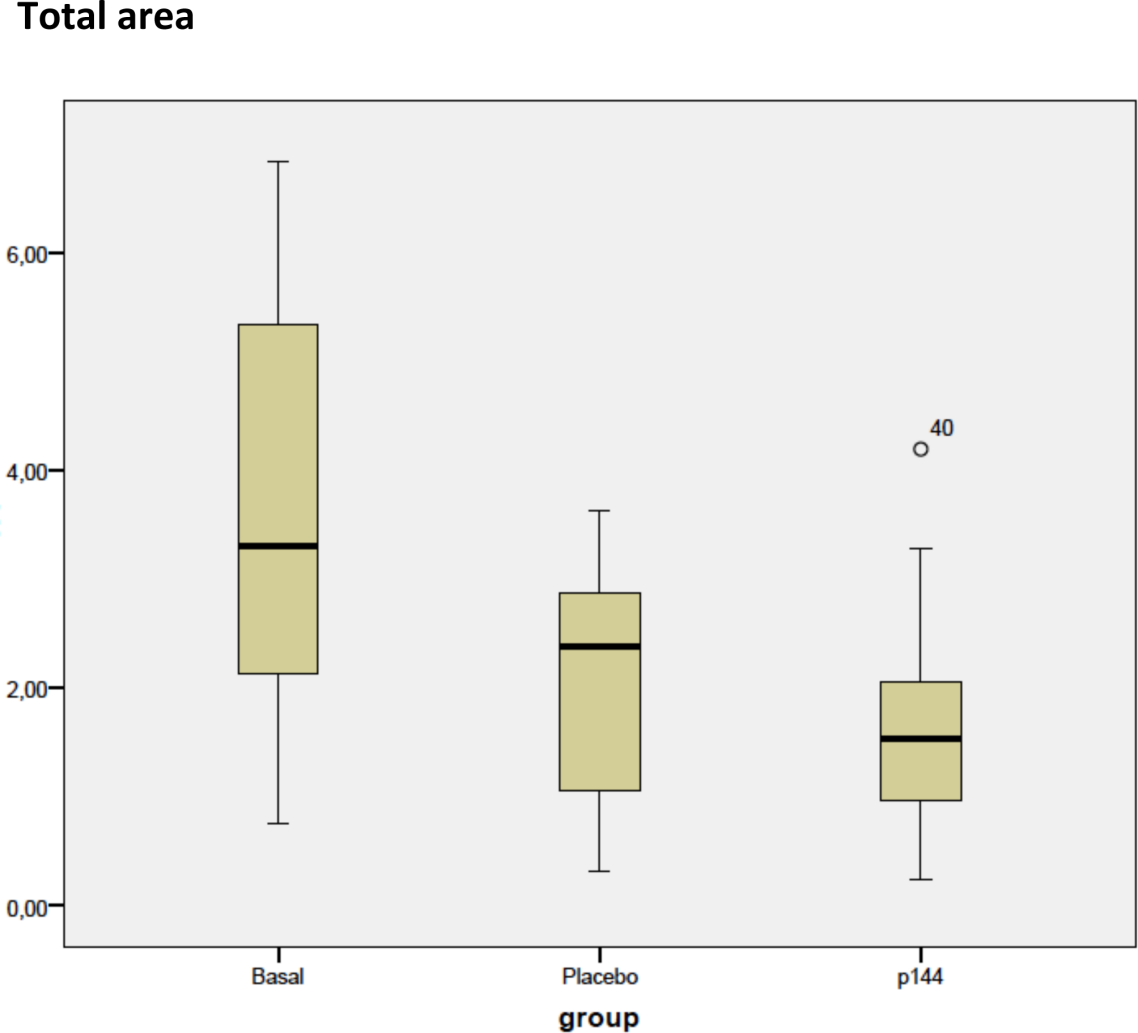
Box plots representing values corresponding to total area of the three groups. (cm2).

In subgroups analyses, the mean values of total area for placebo group (A) was 2.024±1.1 cm^2^ whereas for treatment group (B) it was 1.68±1.01 cm^2^. This difference did not reach statistical significance. (Fig 2)

**Fig 3:**
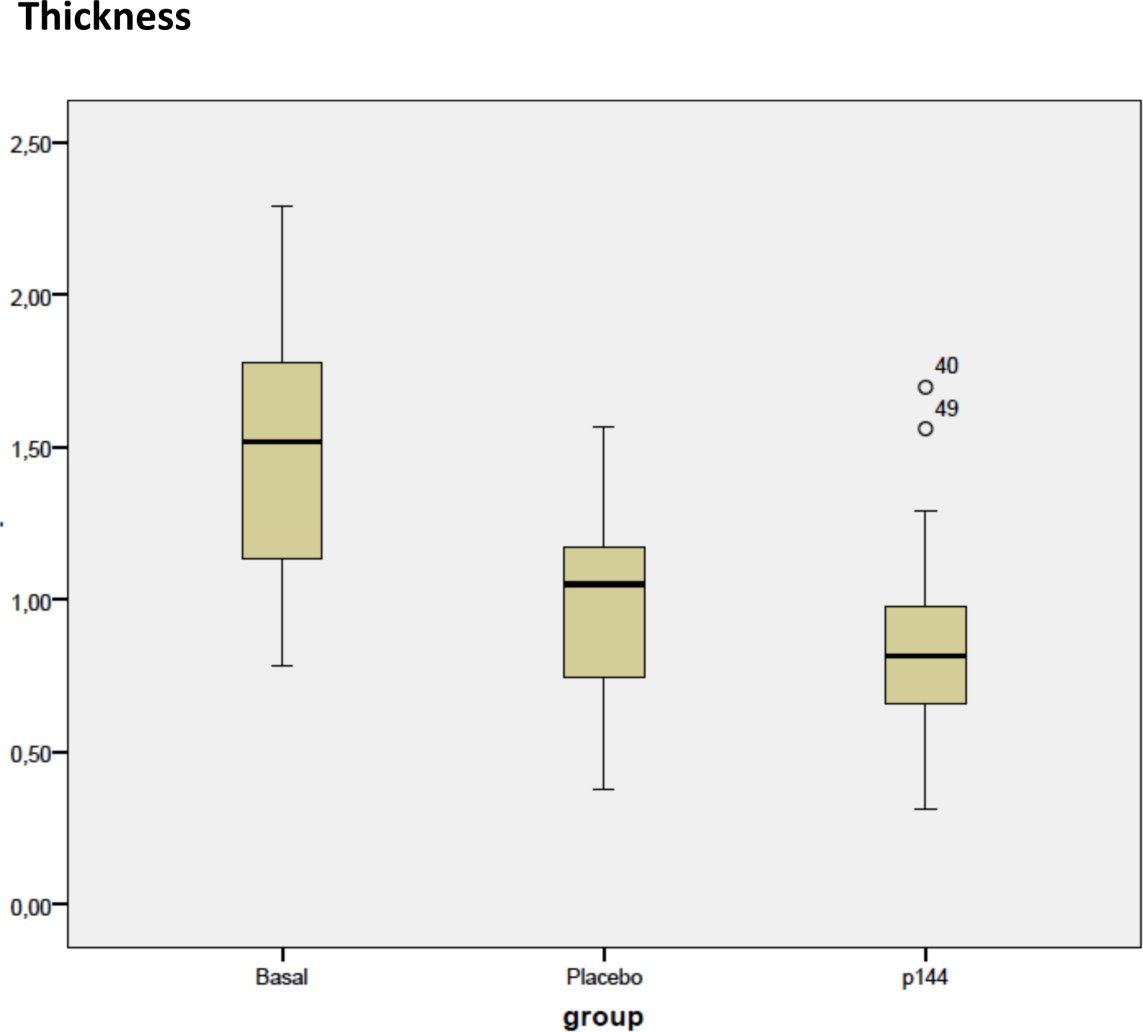
Box plot corresponding to collagen fibers area of the three groups (cm^2^).

The collagen fibers area was measured as area occupied by the collagen fibers relative to total area. The mean area for the placebo group (A) was 1.74±1.1 cm^2^ and for P144 group (B) was 1.5±1.01 cm^2^. There was a trend towards reduction of area in treatment group although this difference did not reach statistical significance (*p* = .306). (Fig 3)

**Fig 4:**
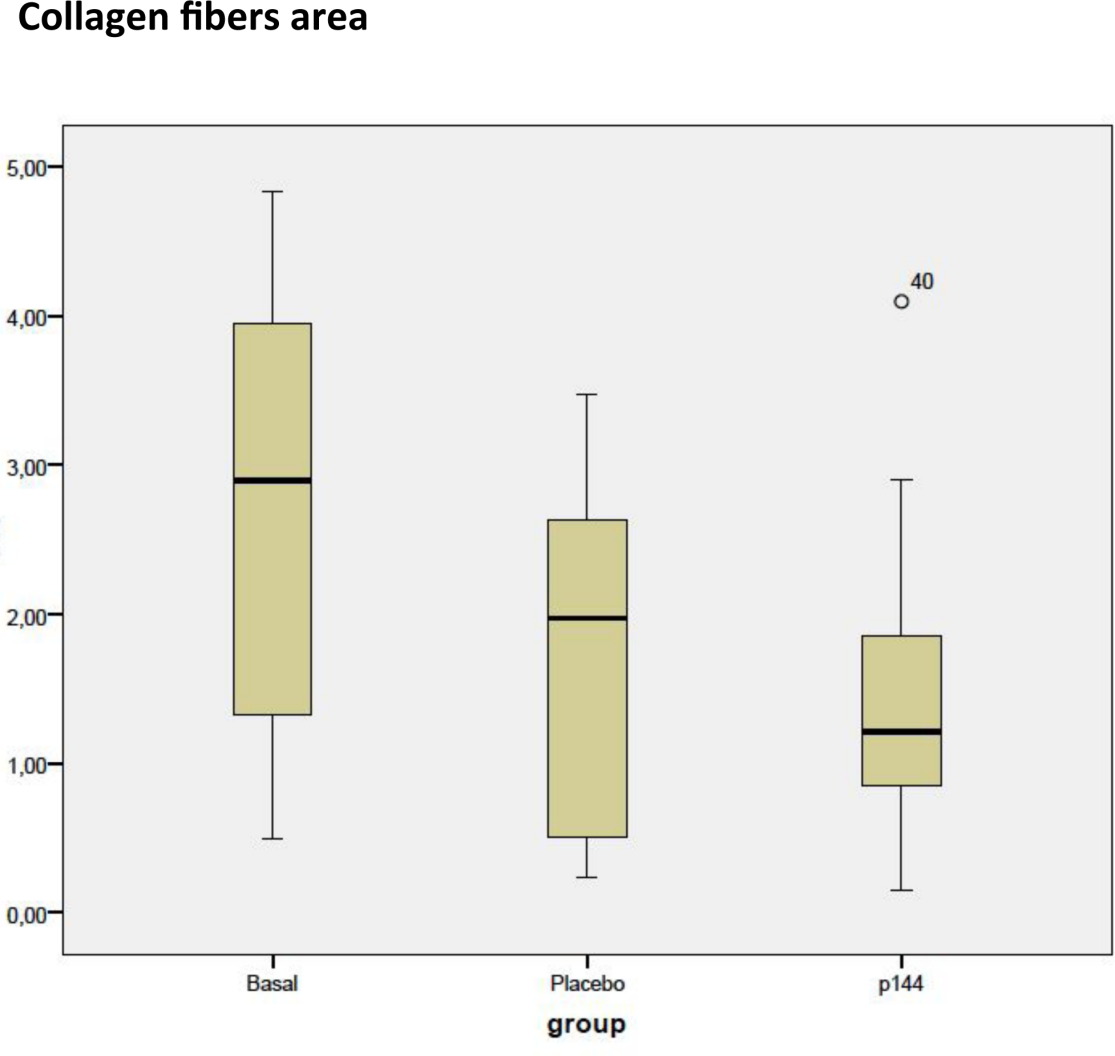
Box plot corresponding to thickness of scars in three groups (cm).

The mean values of thickness of the scars for placebo group was 0.99±0.35 cm whereas for treatment group it was 0.88±0.36 cm. This difference was not statistically significant but a trend was observed in the reduction of thickness in the treatment group. In the subgroups comparison, both group A and group B in comparison with basal group (C) showed statistical difference in reduce area occupied by collagen fibers and thickness. (Fig 4)

**Fig 5:**
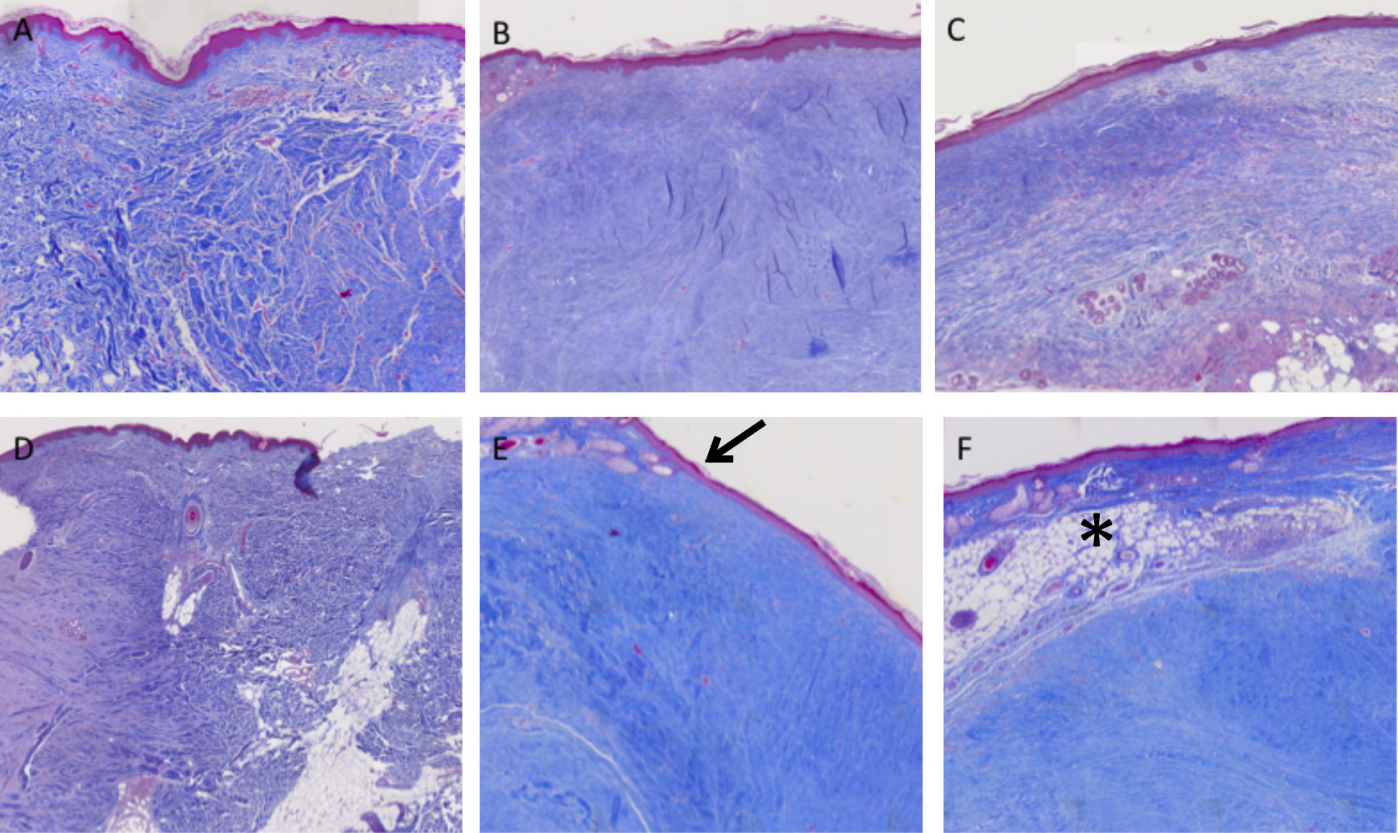
Histological preparations of hypertrophic scars staining with Masson’s Trichrome. (20x magnification).

In Fig 5 two examples are shown providing basal group, placebo group and treatment group.

A. Basal group (no implanted scar). Intense dermal collagen proliferation with thickened and rearranged collagen bundles. Epidermis is maintained with normal features and absence of skin appendages is observed; B. Same scar of A, after excision from the mouse, placebo group. Very intense dermal collagen proliferation with disarrangement of the collagen fibers. Epidermis resembles human epidermis with absence of skin appendages; C. From same scar, treatment group. Moderate dermal collagen proliferation with normal human epidermis; D. Basal group. Hypertrophic scar with very intense dermal collagen proliferation and engrossed, disarranged collagen bundles; E. Placebo group. Moderate dermal collagen proliferation with human epidermis preserved. Transition between mouse and human skin is visible (black arrow); F. Scar from treatment group, with moderate dermal collagen proliferation and human epidermis features. In this case the scar was partially covered by mouse skin which is provided with many hair follicles. (Black star)

### Changes in the collagen I, collagen III and fibrillin-1

**Fig 6:**
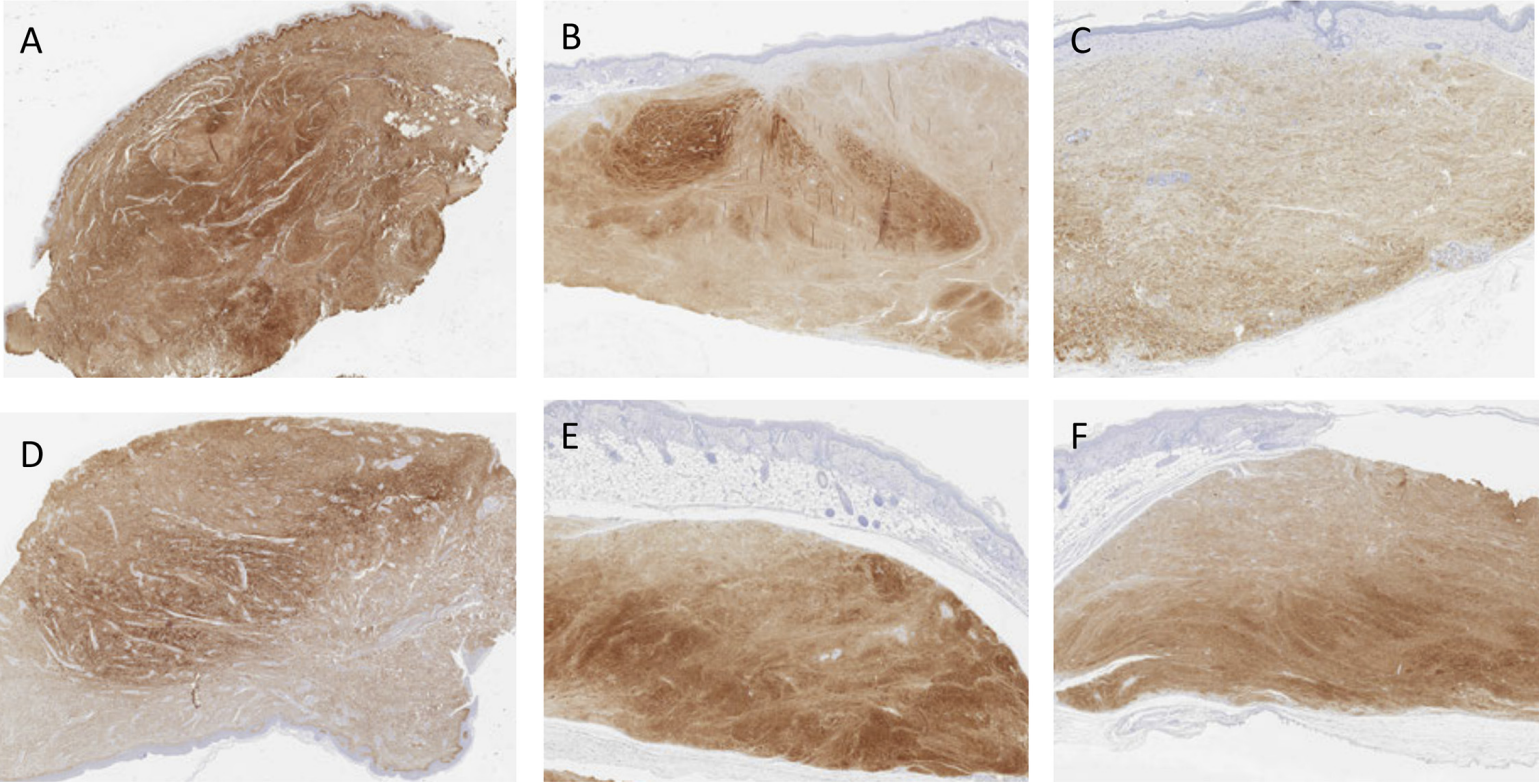
Images of hypertrophic scars staining with anti-collagen I. A (basal), B (placebo) and C (treatment) belong to the same scar. D (basal), E (placebo) and F (treatment) are from a second scar. Fewer stained fibers can be seen in C and F when comparing with the other groups.

**Fig 7:**
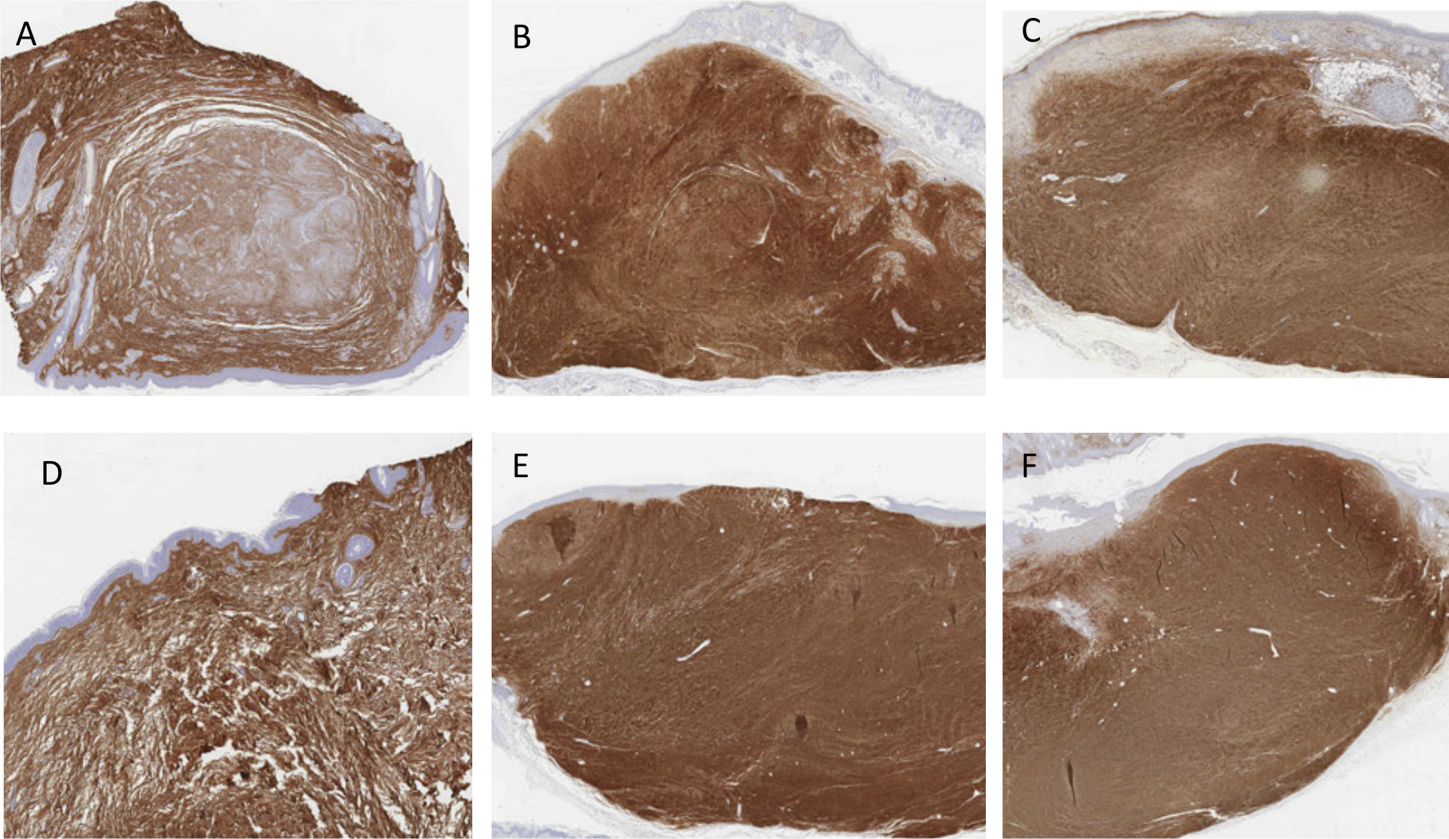
Images of hypertrophic scars staining with anti-collagen III. A (Basal), B (placebo) and C (treatment) represent the same scar. Differences between these three groups is minimal. Images from a second scar, D (basal), E (placebo) and F (treatment).

**Fig 8:**
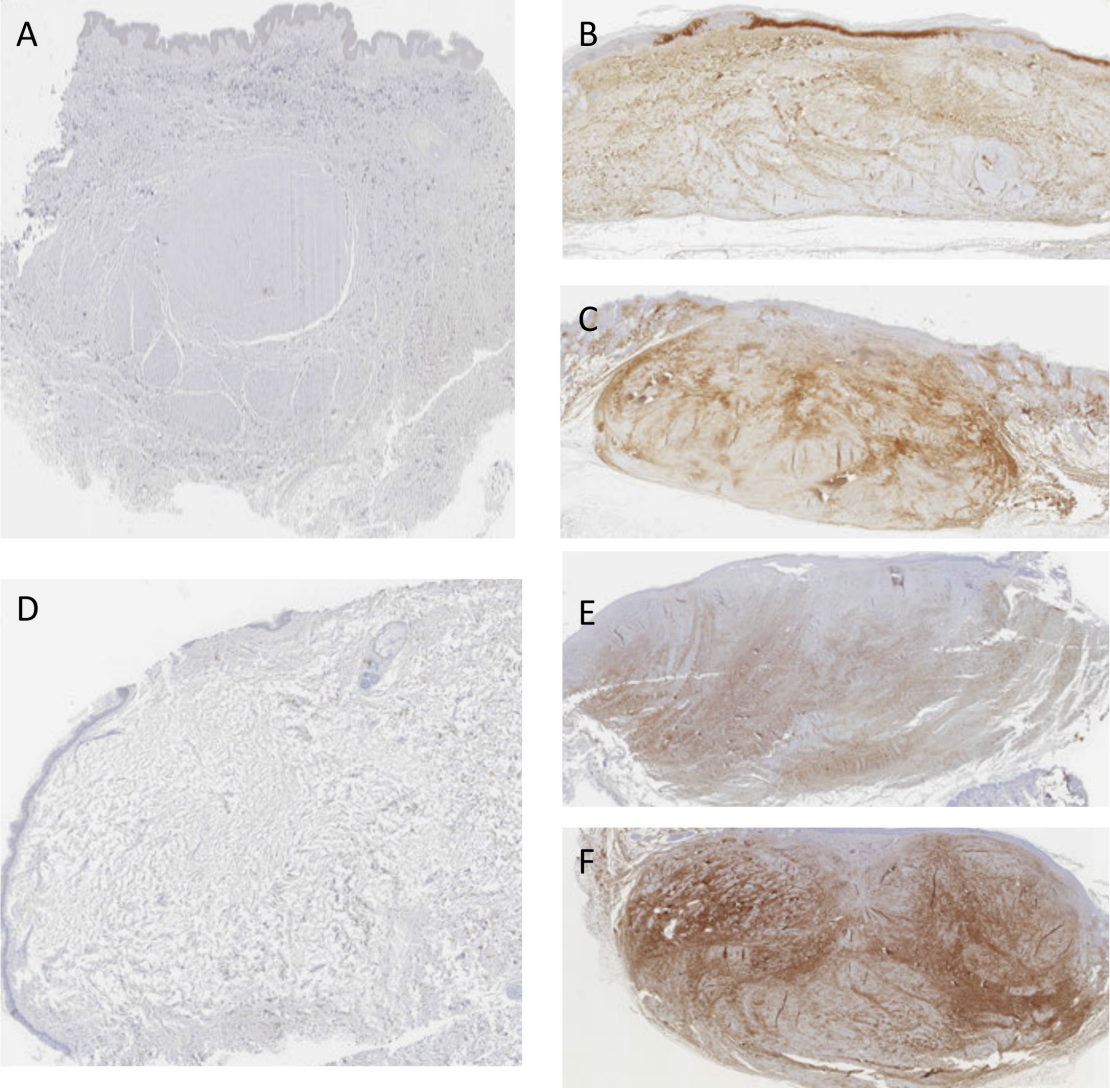
Images of hypertrophic scars staining with anti-fibrillin-1. A (basal), B (placebo) and C (treatment) belong to the same scar and D (basal), E (placebo) and F (treatment) to a second one. It is noticeable that there is almost a complete absence of elastic fibers in A and D (basal group). Rearrangement of elastic fibers system is more obvious in pictures C and F.

Sections of the scars were stained with anti-collagen I, anti-collagen III and anti-fibrillin-1 antibodies (Figs 6–8). Statistical reduction of Collagen I expression and increase of Fibrillin-1 staining in the implanted scars compared with the basal group was found (Tables 3 and 4). The differences of Collagen III in the three groups did not reach statistical significance. Proportional changes were found in the collagen deposition since the ratio collagen I/Collagen III was maintained. See S2 Table.

**Table 3:** Comparison between the groups of collagen I, collagen III, Fibrillin-1 and ratio Collagen I/ Collagen III.

Variable	Group	N	Mean	SD^1^	SME^2^
Collagen I	Basal	18	70.16	16.44	3.87
(Ratio%)	Placebo	18	49.72	20.84	4.91
	P144	18	50.5	20.88	4.92
Collagen III	Basal	18	67.5	23.88	5.63
(Ratio %)	Placebo	18	61.8	24.08	5.67
	P144	18	58.5	20.06	4.73
Col I/Col III	Basal	18	1.82	2.75	0.64
	Placebo	18	1.14	1.32	0.31
	P144	18	1.18	1.34	0.32
Fib-1	Basal	18	1.88	2.587	0.61
(Ratio%)	Placebo	18	27.33	16.26	3.83
	P144	18	32.77	17.15	4.04

^**1**^Standard deviation

^**2**^Standard mean error

**Table 4:** Statistical results in the comparison between subgroups.

Variable	P144 Vs. Placebo	Basal Vs. P144	Basal Vs. Placebo
Collagen I	.912	.003	.002
Collagen III	.649	.229	.488
Ratio Col I/Col III	.943	.379	.355
Fib-1	.335	.000	.000

Figs 6–8 show some examples of scars staining with anti-collagen I, anti-collagen III and anti-fibrillin-1.

In the subgroups analyses, no statistical difference was found comparing P144 group with placebo in any variable. However, a trend of lower levels of collagen III was found in the treatment group. Fibrillin-1 was higher in treatment group although the difference did not reach statistical significance. Significant difference was resulted when comparing basal group vs P144 group in the collagen I (p = .003) and in fibrillin-1 expression (p = .000). Similar results were found when comparing basal group with placebo group (p = .002 and p = .000 respectively).

## Discussion

The “in vivo” model obtained by implantation of human hypertrophic scars implanted onto nude mice retained the morphology, histotypic characteristics and viability of human scar for more than 50 days, according to our study. This finding was supported by previous studies developed by Shetlar [19]. Study of hypertrophic scarring has been challenging from the beginning given that most of the experimental animals do not develop keloid or hypertrophic scars [20]. This has constituted the main limitation of using animals to study the pathogenesis of fibrotic processes [21]. To overcome this problem fibroblast isolated from some of fibrotic disorders were cultured in order to extensively examine the physiological changes occurred during abnormal scarring [20,22]. However, the results of such studies can only provide insight into the pathogenesis of cutaneous fibrosis but they could not fully resemble the actual processes of hypertrophic scars in humans. For instance, the expression mRNA of the proteins can accurately be determined in these studies but could not be correlated to a truly deposition of these proteins in the scars. Although mechanisms behind pathogenesis can be study in depth with this method, reproducing a more holistic method that may resemble the conditions leading to abnormal healing could not be accomplished with isolated fibroblasts.

For decades, “in vivo” models of human hypertrophic scar have been used to study their behavior or to test new therapies. These “in vivo” models can be obtained by direct implantation of human tissue samples [23], either hypertrophic scars or skin grafts [24] or using scientific artifices to cause hypertrophic scarring. Many examples of these artificial mechanisms have been described, such as burning in large white pigs [25], creating a split thickness wound in red duroc guinea pigs [26], rabbit ear model [27] or administration of subcutaneous bleomycin in nude mice to stimulate dermal proliferation. The main limitation of these models was the inability to reproduce normal process of hypertrophic scar development [28]. Moreover, basal differences may bias studies results seen as abnormal scarring may develop differently in each animal. Similarly, when using direct transplantation of clinical hypertrophic scar into animals, this scar may also not contain the initiating factors that originally led to the development of the disease. In spite of well-known limitations of an “in vivo” model, implantation of human hypertrophic scars into nude mice exhibits many similarities and few disparities with hypertrophic scars in patients [29]. Indeed, the same level of glycosaminoglycan levels were maintained in the transplanted tissue samples for at least 60–80 days [19]. We observed in this study that those implanted human scars, either from placebo group or treatment group showed a reduction in total area, collagen fibers area and thickness of the scars. Probably this finding may respond to a similar behavior encountered in human skin grafting, with a trend to shrink or contracture after the shedding period [30]. Results from the study of Kischer et al. [31] supported this possibility given that they found a volume reduction in the implanted hypertrophic scars and keloids in mice. According to the obtained results in this study, reduction of total area encountered in P144 group was higher than in placebo, more than 17%. Although it did not reach statistical significance, this change should have clinical impact. Probably greater changes were needed to reach statistical significance to overcome this phenomenon that happened in both placebo and treatment group.

Progressive scar maturation after implantation in mice was evidenced by a decrease in collagen I and an increase in fibrillin-1 expression. Previous studies have shown that elastic fibers were absent in the immature scars and began to reappear at the end of the remodeling and beginning of the resolution phase [32,33]. The expression of fibrillin-1 detected in P144 treated group was the highest within the three groups, with an increase of 94% compared with basal group. Re-establishment of elastic fibers system may partly contribute to reduction in total area of scars in treatment group. Decrease in collagen I could be explained because of collagen breakdown and rearrangement during scar maturation process [28]. Similarly, production of collagen III was not expected to vary significantly in any of the groups. The synthesis of collagen III was more prominent in the early phases of the wound healing whereas at the final phases it was replaced by stronger fibers of collagen I [23]. For this reason expression of collagen III remained unchanged during the study period. A decrease in collagen content was expected with the treatment however, according to previous authors, this was difficult to achieve. These authors [34] measure amount of collagen in post-burned scars in different time intervals, from 1, 3, 6, 12, 18 and 24 months. No differences in the amount of collagen were found when comparing scars of 18–24 months with scars at earlier time points. They concluded that synthesis and catabolism of collagen might be in balance at 18 months but an excess of collagen remained. In a previous report, activation of collagenase [35], a type of matrix metalloproteinase induced initiation of collagen degradation. However, in case of hypertrophic scars, its fibroblasts expressed much less mRNA collagenase than normal fibroblasts. In the light of these previous studies, it was unlikely that with inhibition of TGF-β1 activity collagen content will decrease, due to the aforementioned evidence and additionally, because downregulation of TGF-β1 did not promote collagenolysis or collagen breakdown directly [12,36]. Scarce studies had dealt with ratio of collagen I/collagen III in hypertrophic scars. For normal scars this ratio was 3:1 or 6:1 whereas for keloids this was 15:1 or 17:1 [37]. However, for hypertrophic scars this ratio was not determined and should not be considered as an intermediate value between the previous entities. The basal group of the current study presented a ratio of 1.8:1, which did not resemble to either normal scars or keloid scars. Probably, with progression of healing process, collagen III would be replaced by collagen I and this ratio will return to normal.

The present study was designed for two-weeks treatment period due to the following: (1) previous “in vivo” models using nude mice were conducted with limited durations, commonly from several hours to few months [38–41]. According to Brayton et al. [42], chronic studies could increase the incidence of neoplasms, glomerulosclerosis and cardiac calcinosis in nude mice. Additionally, many factors may influence experimental outcomes in longer studies such as the diet, housing conditions, infectious agents, light cycle and temperature [42]; (2) morphological features of human hypertrophic scars were maintained for 60 to 80 days after implantation [19]. After that period, the scar may loose some of its characteristic features and not resemble a human hypertrophic scar and finally, (3) on the basis of the study of Santiago et al. [17] it seems that two weeks were a time long enough to see the first changes in a scleroderma model in C3H mice. They topically applied P144 lipogel in combination with subcutaneous bleomycin. Achieved improvement in the skin fibrosis and decrease in soluble collagen content was observed after two weeks. Further supporting this design, another study conducted by Zhao et al. [10] tested the efficacy of transdermal patch with an inhibitor of TGF-β1 and significant results in lower levels of TGFβ1 protein were observed at two weeks after treatment.

Among the studies using P144 and other types of inhibitor of TGF-β receptors for different fibrotic condition, very few of them used topical application alone and the results measured consisted on the changes in the signaling or expression of cytokines [43], extracellular matrix components [44] or regulatory genes [45]. Only few studies using topical or non-invasive therapies in animal models measured morphological and histological features of the scars after treatment. Two of them were aimed out by Aksoy [46] and Brown [47]. In the first study, the authors applied topical zinc oxide in rabbit ear model during three and six weeks and at the end point, the treatment resulted in clinically decreased hypertrophy in the scar. In the second study, reduction of scar volume was achieved upon exposure with high-dose UV light of 7 to 14 days in a rabbit ear model. Although rabbit ear scars were universally accepted as a good reproduction of fibrotic status, these studies were not conducted in human hypertrophic scars as in the current study. In a similar study, Santiago et al. [43] combined the topical application of P144 during 2 weeks with four weeks of subcutaneous injection of bleomycin in a murine model of scleroderma. The authors found a suppression of the expression of CTGF, α-SMA, without modification in cell proliferation. However, the former study did not showed whether morphological changes were observed after treatment. Querceptin [48], which inhibits the expression of TGF-β1 receptors type I and II, was used as topical treatment but the results were not conclusive to demonstrate its efficacy. When treating patients, the goal should be a decrease in the volume and height of the scars, with clinical improvement. Consequently, outcomes included should mainly base on the measureable macroscopic changes, with histologic and morphological correlation.

Recently, polytherapy for treatment of hypertrophic scars has been used in patients with favorable results. Combination of triamcinolone and 5-fluor-uracilo, lasertherapy and injection of corticosteroids [49], silicon plate with addition of vitamin E [50] seem to be promising to achieve better results than using them separately. Following this idea, probably the effect of the topical application of P144 may be enhanced in addition of a second agent, which can induce collagenolysis and cell apoptosis. Pulsed-dye laser [51], silicone-based products [52] and pressure dressings [53] can induce fibroblasts apoptosis and increase the collagen degradation. A combined drug therapy may yield greater efficacy and reduce side effects [54].

Non-invasive therapies are the preferred choice for hypertrophic scars treatment because these require a long period of treatment until some improvement can be achieved. Moreover, topical application could facilitate therapeutic adherence, which is paramount to achieve good results.

The topical application of P144 within a two-week period of treatment has provided promising results in terms of scars maturation and scars macroscopic features. Further studies were necessary to elucidate whether the application of P144 in the early phase of the hypertrophic scar formation might avoid the excessive deposition of extracellular matrix components, hence as a preventive treatment.

## Supporting Information

S1 Table Results of the measurement of the total area, collagen area and thickness in all cases included in the study. (PDF)

S2 Table Results of the measurement of the expression of Collagen I, Collagen III and the ratio Collagen I/III in all cases included in the study. (PDF)
