# Supplementary material for: Effect of P144® (Anti-TGF-β) in an “In Vivo” Human Hypertrophic Scar Model in Nude Mice
Source: PLoS One. 2015 Dec 31;10(12):e0144489. doi: 10.1371/journal.pone.0144489 (PMC4697841; doi:10.1371/journal.pone.0144489)
Supplement: S1 Table — (PDF) [file pone.0144489.s001.pdf]

CASES	Total Area	Collagen Area	Thickness
1 Basal	3,959	2,289	1.515
1 Placebo	1.853	1.001	1.092
1 p144	1.153	0.995	0.695
2 Basal	6.035	4.11	2.016
2 Placebo	2.648	2.266	1.221
2 p144	1.301	0.785	0.862
3 Basal	4.423	3.117	1.404
3 Placebo	1.35	0.82	1.049
3 p144	1.941	1.675	1.08
4 Basal	1.24	1.132	0.923
4 Placebo	2.99	2.752	1.24
4 p144	4.696	4.456	1.789
5 Basal	1.42	0.989	1.243
5 Placebo	0.592	0.517	0.499
5 p144	0.531	0.357	0.411
6 Basal	6.035	4.863	2.042
6 Placebo	3.826	3.543	1.52
6 p144	2.941	2.204	1.341
7 Basal	7.159	4.609	2.413
7 Placebo	1.233	1.162	0.839
7 p144	1.276	0.26	0.859
8 Basal	5.343	4.711	1.816
8 Placebo	3.408	3.072	1.359
8 p144	2.246	1.677	1.268
9 Basal	4.935	3.512	1.784
9 Placebo	2.893	2.369	1.305
9 p144	1.674	1.22	0.914
10 Basal	2.853	2.487	1.188
10 Placebo	0.723	0.466	0.645
10 p144	1.158	1.012	0.841
11 Basal	6.543	4.469	2.028
11 Placebo	4.045	3.63	1.731
11 p144	4.504	3.955	1.529
12 Basal	4.787	3.401	1.792
12 Placebo	3.54	3.127	1.102
12 p144	1.12	0.942	0.703
13 Basal	0.876	0.671	0.809
13 Placebo	1.467	1.155	1.049
13 p144	6.082	4.786	2.483
14 Basal	3.979	3.206	1.336
14 Placebo	0.334	0.299	0.405
14 p144	0.327	0.192	0.329
15 Basal	3.293	2.114	1.565
15 Placebo	2.224	1.908	1.118
15 p144	3.04	2.853	1.369
16 Basal	1.192	0.832	1.081
16 Placebo	2.899	2.74	1.146
16 p144	2.837	2.133	1.173
17 Basal	1.962	1.523	1.008
17 Placebo	2.246	1.997	1.069
17 p144	1.704	1.342	1.073

18 Basal	0.805	0.498	0.843
18 Placebo	0.903	0.496	0.681
18 p144	0.627	0.332	0.627
