# Supplementary material for: Effect of P144® (Anti-TGF-β) in an “In Vivo” Human Hypertrophic Scar Model in Nude Mice
Source: PLoS One. 2015 Dec 31;10(12):e0144489. doi: 10.1371/journal.pone.0144489 (PMC4697841; doi:10.1371/journal.pone.0144489)
Supplement: S2 Table — (PDF) [file pone.0144489.s002.pdf]

CASES	Collagen I	Collagen III	Fibrillin-1
1 Basal	69.68	40.87	2.34
1 Placebo	83.81	19.87	19.83
1 p144	55.64	69.23	14.43
2 Basal	87.65	82.22	5.88
2 Placebo	34.17	45.12	36.28
2 p144	55.54	10.59	8.07
3 Basal	46.69	73.91	1.10
3 Placebo	64.17	73.10	45.10
3 p144	75.30	93.74	2.26
4 Basal	81.26	7.88	1.30
4 Placebo	51.56	86.16	41.79
4 p144	41.92	94.43	55.01
5 Basal	57.12	72.52	1.29
5 Placebo	78.76	15.04	56.35
5 p144	62.62	77.49	19.47
6 Basal	53.94	64.43	2.65
6 Placebo	48.78	60.71	43.30
6 p144	46.98	49.76	31.94
7 Basal	88.47	84.30	0.07
7 Placebo	58.11	71.06	47.51
7 p144	73.98	84.18	66.09
8 Basal	48.22	76.12	0.77
8 Placebo	35.56	52.91	37.77
8 p144	14.82	55.99	17.35
9 Basal	62.83	80.74	0.18
9 Placebo	48.39	54.97	28.84
9 p144	74.74	20.48	35.15
10 Basal	81.35	13.09	0.28
10 Placebo	59.99	46.38	39.44
10 p144	40.25	50.24	44.37
11 Basal	84.90	86.33	0.19
11 Placebo	44.16	77.43	33.67
11 p144	29.38	67.38	18.26
12 Basal	86.29	86.96	0.87
12 Placebo	74.00	70.67	18.21
12 p144	76.98	76.49	30.14
13 Basal	74.57	84.66	0.98
13 Placebo	59.70	79.69	64.72
13 p144	69.96	73.83	31.45
14 Basal	83.28	76.72	2.91
14 Placebo	20.90	41.17	1.06
14 p144	23.89	25.70	30.88
15 Basal	89.46	73.75	2.82
15 Placebo	26.85	60.29	27.99
15 p144	19.57	62.94	9.56
16 Basal	68.31	81.65	9.30
16 Placebo	3.50	53.60	5.70
16 p144	25.52	77.49	31.70
17 Basal	71.70	81.55	0.60
17 Placebo	59.59	79.92	9.31
17 p144	60.77	79.79	21.42

18 Basal	36.09	57.79	2.53
18 Placebo	66.96	73.37	42.60
18 p144	59.37	54.25	31.82
